# A Nafion Film Cover to Enhance the Analytical Performance of the CuO/Cu Electrochemical Sensor for Determination of Chemical Oxygen Demand

**DOI:** 10.3390/s19030669

**Published:** 2019-02-06

**Authors:** Tanya Carchi, Byron Lapo, José Alvarado, Patricio J. Espinoza-Montero, Jordi Llorca, Lenys Fernández

**Affiliations:** 1 Grupo BIOeng, Escuela de Ingeniería Química, Universidad Técnica de Machala, Apartado 070151, Machala, Ecuador; tanyacarchi@gmail.com (T.C.); blapo@utmachala.edu.ec (B.L.); 2 Departamento de Química, Universidad Simón Bolívar, Apartado 89000, Caracas, Venezuela; jalvar@usb.ve; 3 Pontificia Universidad Católica del Ecuador, Escuela de Ciencias Químicas, Avenida 12 de octubre y Roca, Apartado 17-01-2184, Quito, Ecuador; 4 Escuela Politécnica Nacional, Escuela de Formación de Tecnólogos, Apartado 17-01-2759, Quito, Ecuador; 5 Institut de Tècniques Energètiques and Centre for Research in Nanoengineering, Universitat Politècnica de Catalunya, Barcelona 08034, Spain; jordi.llorca@upc.edu

**Keywords:** chemical oxygen demand, copper oxide electrode, nafion film, detection

## Abstract

We modified and evaluated the performance of a CuO/Cu electrochemical electrode for chemical oxygen demand (COD) determination by covering it with a Nafion (Nf) film. The resulting modified CuONf/Cu electrode sensor was used for the electrochemical determination of COD in river, slaughterhouse and estuarine water samples in order to evaluate its performance for this particular task. It was compared with the CuO/Cu sensor with no Nafion. The main electrochemical characteristics of interest, resistance, sensitivity, accuracy and reproducibility, were assessed by means of Linear Sweep Voltammetry using glucose as a standard. Results of these essays indicate that the procedure used produced smooth and firmly attached Nf films covering the whole copper surface. This sensor was shown to be resistant to interferences and effective in electro-oxidation of a wide range of organic compounds and therefore very useful for COD determination. Using the newly developed CuONf/Cu electrode an analytical linear range of 50 to 1000 mg·L^−1^ COD, with a detection limit of 2.11 mg·L^−1^ (n = 6) COD was achieved. The comparison shows that the CuONf/Cu sensor is more appropriate for COD determination than its counterpart with no Nafion.

## 1. Introduction

Oxygen, even though it is barely soluble in water, is an essential element for preserving aquatic life. Without free oxygen, ecosystems cannot support life. Therefore, organic contaminants which consume oxygen during their decomposition could affect, in a fast and direct way, the survival of these ecosystems. The extent of organic contamination which could consume oxygen in aqueous systems could be evaluated by measuring their chemical oxygen demand, COD. This is one of the most important parameters to take into consideration when monitoring surface water quality in lakes, rivers or wastewater. COD values represent the amount of oxygen, mg·L^−1^, consumed in the oxidation process of organic contaminants by oxidant reagents such as O_2_, Cr_2_O_7_^−2^, MnO_4_^−^ and Ce^4+^ [1,2]. Reaction kinetics of the oxidant reagents with some organic or inorganic contaminants affecting the COD values of a given water system is quite slow. As a consequence, with classic methods, such as oxidation by permanganate or dichromate [3], the oxidation efficiency for some compounds is low or inclusive nil. This results in COD determinations which are not representative of the real contamination of the samples being analyzed. To overcome this drawback, it is necessary to do more research aiming at improving oxidation reaction kinetics. On top of increased oxidation capacity leading to increased reaction kinetics, the new methods for COD detection should be faster, less laborious, more sensitive and accurate, less expensive, freer from interfering inorganic ions such as chloride and greener than the conventional methods. Several researchers have dedicated effort and time to develop better methods than the conventional ones to determine COD. Ultraviolet spectroscopy [4], fluorescence spectroscopy [5], and chemiluminescence methods [6,7] have been proposed for COD determination. However, the need for digestion steps using strong acids or some other toxic reagents included in these methods could become a safety hazard. A better possibility would be electrochemical oxidation using redox systems with standard potentials higher than those of oxygen, dichromate, permanganate or cerium, i.e., E^0^ > +1.61 V. Electrochemistry methods of analysis are becoming more and more attractive for COD quantitation because they could be simple, accurate, sensitive and safe [8,9,10,11,12,13,14,15,16,17,18,19,20,21]. Ideally, the best method for COD determination should electro-oxidize the organic contaminants to completely transform them into CO_2_ plus H_2_O. But, achieving effective full oxidation of organic contaminants to carbon dioxide and water is not a simple matter and although the oxidation achieved by electrochemical methods performing at high enough voltage is definitely higher than using the conventional methods, it is not possible to get 100% oxidation efficiency. This limitation precludes the use of simple, direct integration of the amperometric wave as a representative measure of the COD content of the sample analyzed. For a real sample, where the amount of species which could be oxidize are numerous and usually unknown, the calculation is imprecise, to say the least. 

According to above, the electrochemical approach for contaminants oxidation requires special electrodes [8,9,10,11,12]. Development of electrodes covered with a cobalt oxide film, diamond electrodes doped with boron, electrodes containing lead oxide, Ti, Rh_2_O_3_ and Ag_2_O/CuO have been reported [13,14,15,16,17,18,19,20,21]. These electrodes offer several advantages for COD determination: i.e., fast analysis, direct collection of analytical signals, better sensitivity, low analysis cost and safe operation. As mentioned before, in spite of the fact that 100% contaminants oxidation is very difficult to reach, getting close to it is a necessary step to improve accuracy and precision in the COD electrochemical determinations. In this sense, copper electrodes have found wide acceptance [22,23,24], based on the fact that Cu in alkaline media acts as a powerful electrocatalyst for oxidation of carbohydrates and aminoacids, which are believed to be the major culprits for COD. It is well known that metal electrodes can experience passivation, which could in turn deactivate the electrode rendering it faulty or partially insensitive. Copper is no exception to this passivation effect. However, this limitation could be superseded by modifying the electrode surface. In the work here presented, we evaluate the use of at CuO/Cu electrode, which has been modified by covering it with a thin Nafion (Nf) film to minimize surface passivation. The new CuONf/Cu sensor keeps the electrochemical oxidation capacity of the original CuO/Cu sensor, which gives it enough sensitivity and the Nf cover provides it with excellent resistance to passivation, which gives it extra freedom from interference and longer durability. The new improved electrochemical sensor was used for COD determination in different types of water. Quantitation was performed by assessing the oxidation current from the standards calibration curves obtained by Linear Sweep Voltammetry (LSV).

## 2. Materials and Methods

### 2.1. Reagents

Nafion, (5% (*w*/*w*)), was purchased from Aldrich (St. Louis, MO, USA); dimethyl formamide (DMF) and ethanol, 99.8% from Sigma-Aldrich (St. Louis, MO, USA); NaOH, 99%; glucose, 99.95%; citric acid, lactose, ascorbic acid, and phenol from Merck (Galloping Hill Road, Kenilworth, NJ, USA); CuSO_4_·5H_2_O and H_2_SO_4_, 95–98% from Mallinckrodt AR (Dublin, Ireland). All reagents used were analytical grade reagents or better unless otherwise stated. Aqueous solutions were prepared using distilled/deionized, 18 MΩ·cm^−1^, Millipore water.

### 2.2. Instrumentation

To study the electrochemistry characteristics of the modified electrodes a CH-Instruments Potentiostat (Tennison Hill Drive Austin, TX, USA), Model 700D, was used coupled to a conventional 15 mL three electrode reaction cell. The modified electrodes in evaluation (CuO/Cu and CuONf/Cu), were used as working electrodes, a Ag/AgCl electrode was used as reference and a platinum wire (0.5 cm diameter and 3 cm long) was the counter-electrode. Scanning Electron Microscopy (SEM) was performed using a Neon 40 Crossbeam Station (Park Drive, North Billerica, MA, USA) field emission electron source.

### 2.3. Electrodes Fabrication

A copper rod, 3.00 mm diameter and 8.0 cm length, was introduced in a 5.0 mm inner diameter glass cylinder and wrapped with Devcon-2 ton epoxy resin. This electrode was left to dry for 24 h at room temperature. The dry electrode surface was manually polished using 400 and 600 mesh emery paper to minimize surface rugosity, optimize surface exposure and maximize electrical contact between the electrode and the analyte and then the electrode was thoroughly rinsed with distilled water and dried again as it was done before. The bare, dry and clean copper was immersed in a 0.1 mol·L^−1^ NaOH solution submitted to a potential cycling (50 cycles) between −1.0 and +0.80 V vs. Ag/AgCl to obtain the CuO/Cu sensor. For preparation of the CuONf/Cu sensor, five microliters of a 2.5% Nafion/ethanol solution were casted on the polished Cu electrode using a micropipette. Immediately, 3 µL of pure DMF, were added with a micropipette. The ethanol and DMF used as solvents were evaporated by heating at 30 °C with an air gun and rotating the electrode at 50 rpm. The electrode, already covered with a Nafion film (Nf/Cu), was submerged in a 0.1 mol·L^−1^ NaOH solution and submitted to a cycling potential (50 cycles), between −1.0 and +0.80 V vs. Ag/AgCl. This produced the final working version of the CuONf/Cu sensor. 

### 2.4. Analysis of Real Water Samples

The samples were collected from Rio Jubone’s, estuary, from a Huayla inlet and from a slaughterhouse residual water in Machala City, Ecuador. Samples were analyzed by both, the proposed method using the CuONf/Cu electrode and the classical dichromate method [25]. COD electrochemical measurements were performed by linear voltammograms, which were recorded in a potential interval between +0.1 and +1.1 V vs. Ag/AgCl at a scan rate of 80 mV·s^−1^. The working solution was prepared directly in the electrochemical cell by mixing 5 mL of a real water sample with a 0.1 mol·L^−1^ NaOH solution and then homogenizing it with a magnetic stirrer. Before carrying out the measurement the samples were left at rest for around 5 min, and then filtered when necessary. A NaOH solution (0.1 mol·L^−1^) was used to obtain the base current. All experiments were performed at room temperature (~25 °C).

## 3. Results and Discussion

### 3.1. Selection of Electrode Substrate and Modifying Substance

#### 3.1.1. Copper as Substrate 

A number of metals in alkaline media can electrocatalytically oxidize a wide range of organic compounds. We chose Cu as the substrate of our electrodes due to its demonstrated capability as an electrocatalyst for oxidation of the major organic contaminants contributing to COD in water systems, as already mentioned [9]. This Cu catalytic action is mediated by surface oxides, which are easily formed in ambient air or under anodic conditions [22,23,24,26,27,28]. Several studies for the electrocatalytic oxidation of Cu bare electrodes in alkaline media have been carried out using different techniques [29]. This electrochemical process is highly dependent on both the hydroxide concentration and the previous formation of a specific layer of Cu(II) oxide. The participation of Cu(III) species as an electron transfer mediator has been suggested to explain the good performance of Cu electrode in alkaline medium in anodic processes, related to several organic compounds [8,30] in agreement with the following reactions:

Cu+ 2OH^−^ ⇄ Cu(OH)_2_ + 2e^−^

Cu(OH)_2_ + OH^−^ ⇄ Cu(III)OOH^−^ +H_2_O + e^−^

Cu(III)OOH^−^ + Organics_(red)_ + H_2_O ⇄ Cu(OH)_2_ + Organics_(ox)_ + OH^−^

#### 3.1.2. Nafion as the Electrode Modifying Film

We chose Nafion, a cationic exchange polymer formed by perfluorosulfonate branches with sulfonic groups negatively charged at pH > 5 [31], because Nafion is electrochemically inactive, chemically unreactive, hydrophilic, and water insoluble, Nafion forms thin films with a net-like structure which provides a way of selection of contaminants based on their particle size, thus minimizing the possibility of electrode passivation due to adhesion of large contaminant particles on its surface. Additionally, the Nafion film provides the electrode with extra chemical and mechanical endurance. 

#### 3.1.3. Modification of the Electrodes Surface

**Figure 1:**
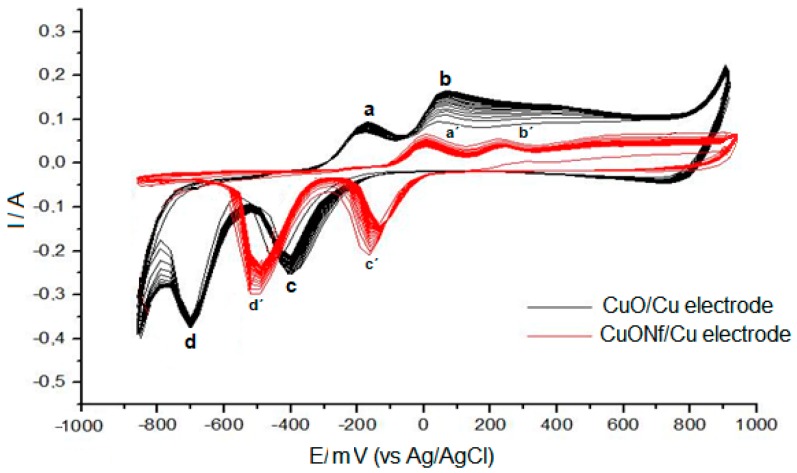
Cyclic voltammograms of CuO electrochemical formation at Cu electrode (black line) and Nf/Cu modified electrode (red line) at 100 mV s^–1^, 50 cycles. Electrolyte solution: 0.1 mol L^–1^ NaOH.

To promote CuO electrochemical formation at the Cu electrode and the Nf/Cu modified electrode, cyclic voltammetry (CV) was employed. Typical responses obtained by cycling the potential (50 cycles) between −1.0 and +1.00 V vs. Ag/AgCl, of the bare copper electrode in a 0.1 mol L^−1^ NaOH, are illustrated in Figure 1 (black line). This massive oxidation resulted in a blackish surface film (formation of copper oxide) on the originally shiny copper surface [8]. The anodic peak at −200 mV corresponds to the formation of a first layer of Cu(I) oxide (Cu_2_O), while the anodic peak at −111 mV corresponds to formation of a second mixed layer of Cu(II) oxide/Cu(II) hydroxide (CuO/Cu(OH)_2_). The signal reaching 800 mV indicates Cu(II)/Cu(III) oxidation. Examining the reverse scan, two cathodic peaks are revealed corresponding to the Cu(II)/Cu(I) (−400 mV) and Cu(I)/Cu(0) (−700 mV) reduction reactions, respectively. Cyclic Voltammetry signals using the Nf/Cu modified electrode in a 0.1 mol·L^−1^ NaOH solution are show in Figure 1 (red line). The response of the Nf/Cu modified electrode is similar to the response of the bare Cu electrode. The waves obtained in the sweep are still present (peaks “a’”, “b’”, “c’” and “d’”, in Figure 1), but show changes in potential to positive values and the peak currents are lower than those obtained using the Cu electrode. This could be explained considering the fact that Nf is a highly insulating material which can act as an inert blocking layer hindering electron and mass transfer. As can be seen in Figure 1, an increase in current from +800 mV at the Cu electrode (black line) and from +900 mV at the Nf/Cu modified electrode (red lines), are practically coincident with the over-potential rise to anodic oxygen formation. It is clear that the CuONf/Cu modified electrode has the highest oxygen over-voltage of the two electrodes under study. This is of advantage for COD anodic determination because the higher the over-potential the lesser the water interference due to oxygen evolution and the more efficient the organic anodic oxidation. 

### 3.2. SEM Characterization of the Modified Electrode

**Figure 2:**
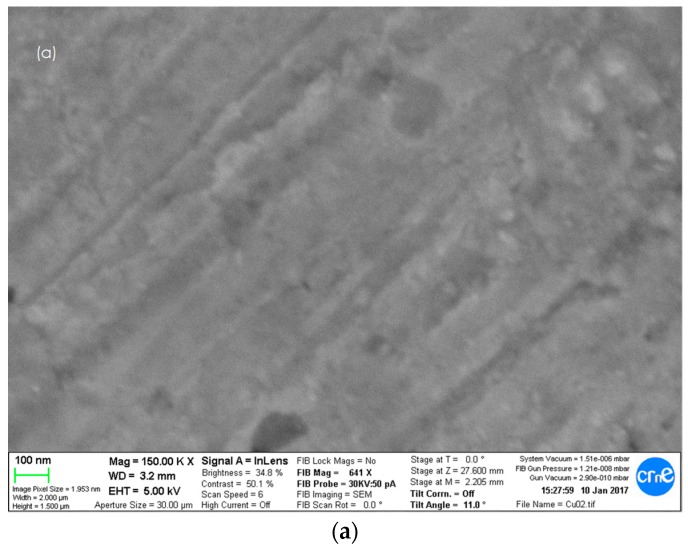

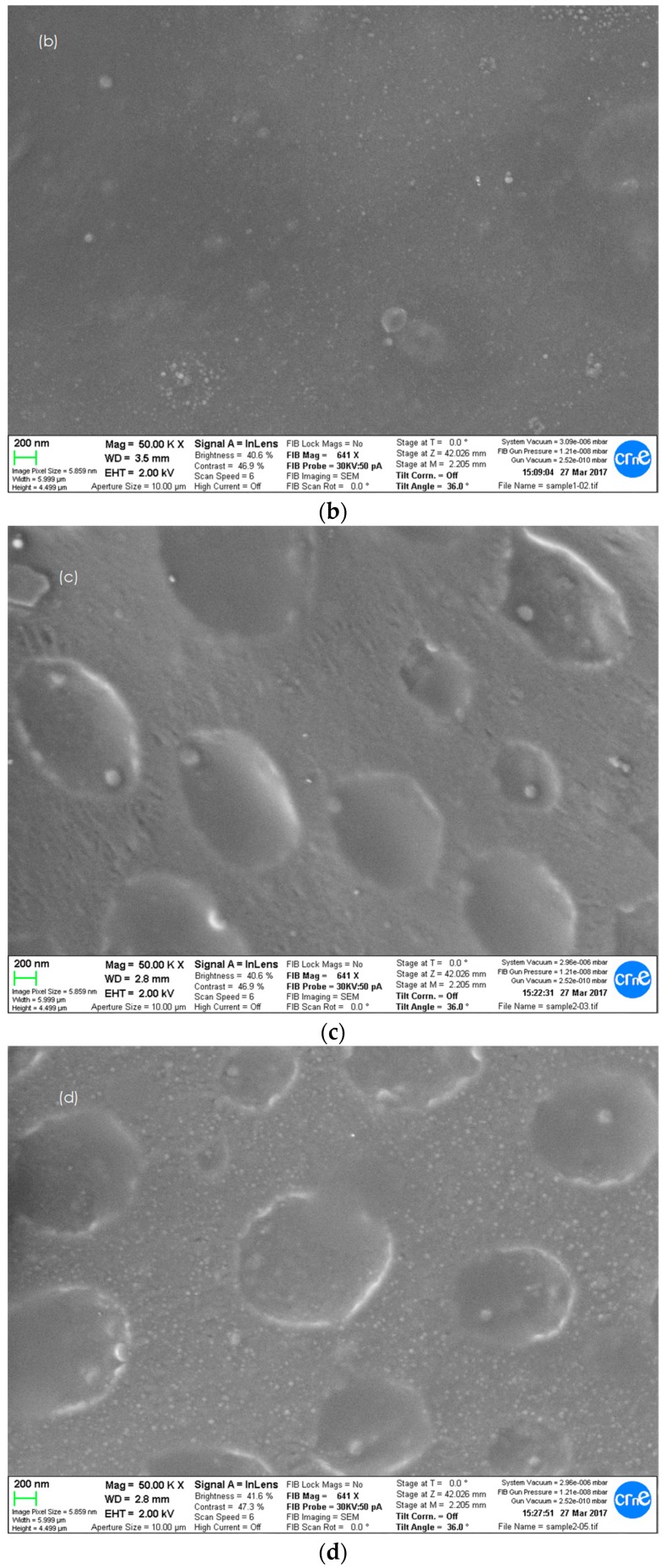

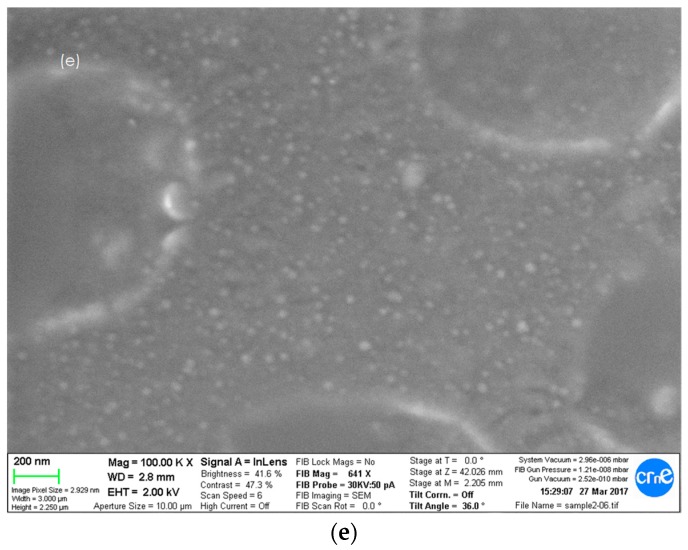
Scanning-Electron-Microscopy of: (**a**) Cu bare electrode; (**b**) CuO/Cu modified electrode; (**c**) Nf/Cu modified electrode; (**d**) CuONf/Cu modified electrode; and (**e**) High magnification SEM images of the CuONf/Cu electrode’s surface.

The surface morphology of the electrodes before and after modification by CV was studied by SEM. Results are shown in Figure 2. The image of the CuO/Cu electrode exhibits CuO particles which appeared to be uniformly spread over the entire electrode surface (Figure 2b). The particles, composed of 30 nm sized granules, look amorphous in structure and shape. The surface of the electrode looks rough and very different from that of the bare Cu (Figure 2a). Nafion forms a film over the substrate’s surface as shown in Figure 2c, with uniformly distributed oval bulges. Figure 2d,e illustrate the low and high SEM image magnifications of the CuONf/Cu electrode obtained after 50 voltammetric Nafion deposition cycles. It shows that the surface is covered by microspheres consisting of nanoparticles, ~36 nm diameter, stretching along different directions on the electrode surface as seen in Figure 2e. Nano-sized channels can be seen to exist between the nanoparticles which could allow for easy diffusion of the electrolyte into the electrode’s surface and out of it.

### 3.3. Electroxidation of Glucose Using the CuO/Cu and CuONf/Cu Sensors

**Figure 3:**
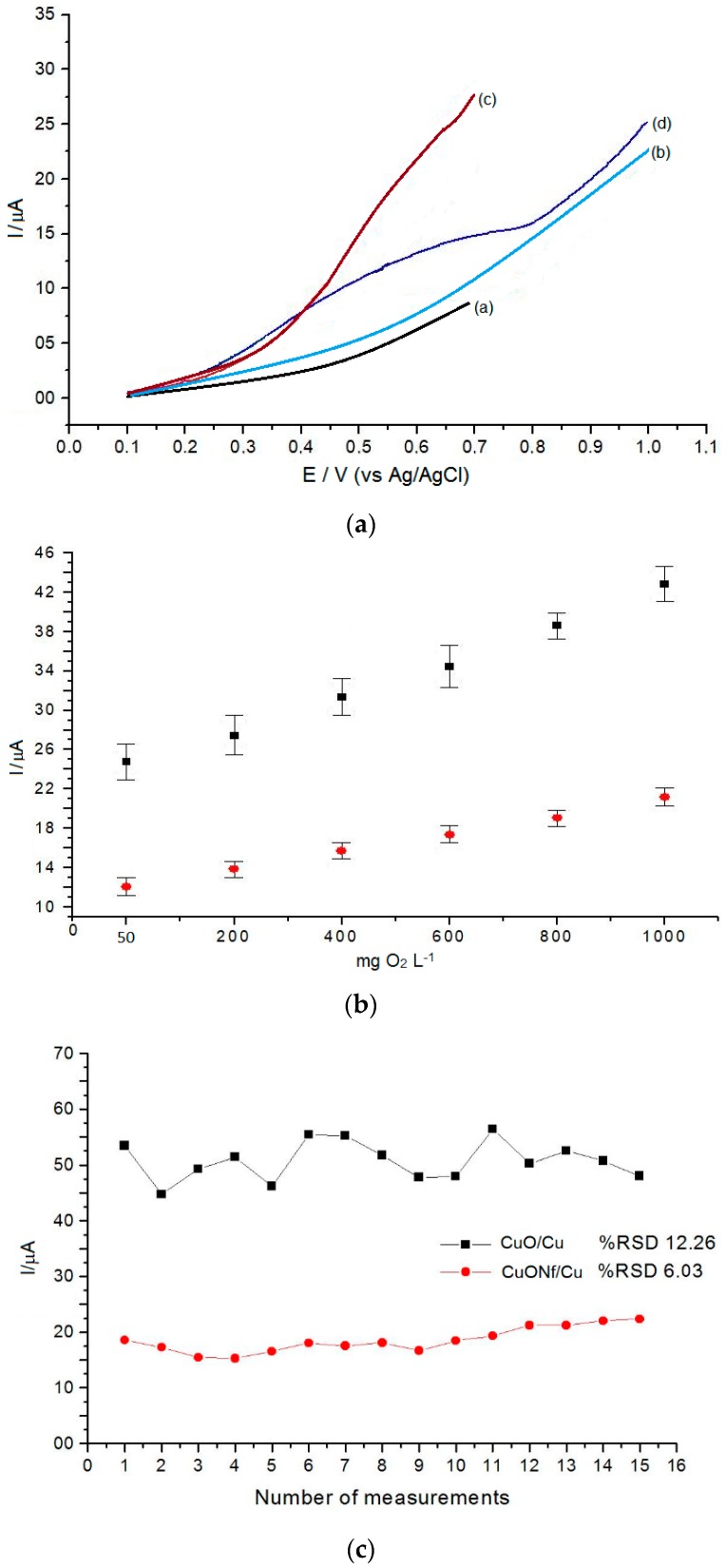
(**A**) Linear sweep voltammograms in 0.1 mol L^−1^ NaOH, without (a and b) and with glucose (COD = 400, mg·L^−1^ of O_2_) (c and d), at: a) Cu/CuO, b) CuONf/Cu, c) Cu/CuO and d) CuONf/Cu electrodes, at 80 mV·s^−1^. (**B**) Working curve Current signal vs. COD concentration (mg·L^−1^), at CuO/Cu (black dots) and CuONf/Cu (red dots) modified electrodes. (**C**) Number of measurements vs. Current signal.

Glucose was used as a standard organic compound for further analytical evaluation of the modified electrodes. Figure 3A, compares the electroxidation behavior of glucose using the CuO/Cu (curves “c”) and using the CuONf/Cu (curves “d”) sensors after linear sweep voltammetry in a 0.1 mol·L^−1^ NaOH solution. Using the CuONf/Cu sensor the oxidation potential of glucose shifts positively, i.e. increases, and the oxidation current decreases as compared to the current using the CuO/Cu sensor. The CuONf/Cu sensor exhibits an enhancement of the potential window for the electrochemical oxidation of glucose, resulting in the decline of water oxidation which otherwise would be the main reaction. It was found that the oxidation current signal of glucose is higher using the CuO/Cu sensor than using the CuONf/Cu modified sensor, but measurements were more reproducible using the CuONf/Cu sensor. It was found that the oxidation current signal at ~0.70 V is proportional to the concentration of COD (C, mg·L^−1^ of O_2_) over the range from 50 to 1000 mg·L^−1^ (Figure 3B). The values of the calibration curves were obtained considering the oxygen concentration consumed in the theoretical oxidation of glucose solutions of exactly known concentration according to C_6_H_12_O_6_ + 6O_2_ ⇄ 6CO_2_ + 6H_2_O. The difference between the theoretical concentration of oxygen produced and the measured oxygen concentration, keeps a linear relationship with the COD values of the sample analyzed. As shown in Figure 3B, the standard deviation of measurements is higher using the CuO/Cu modified electrode, confirming that reproducibility of the measurements with the CuO/Cu electrode is worse than using the CuONf/Cu electrode as visually appreciated in Figure 3A.

**Figure 4:**
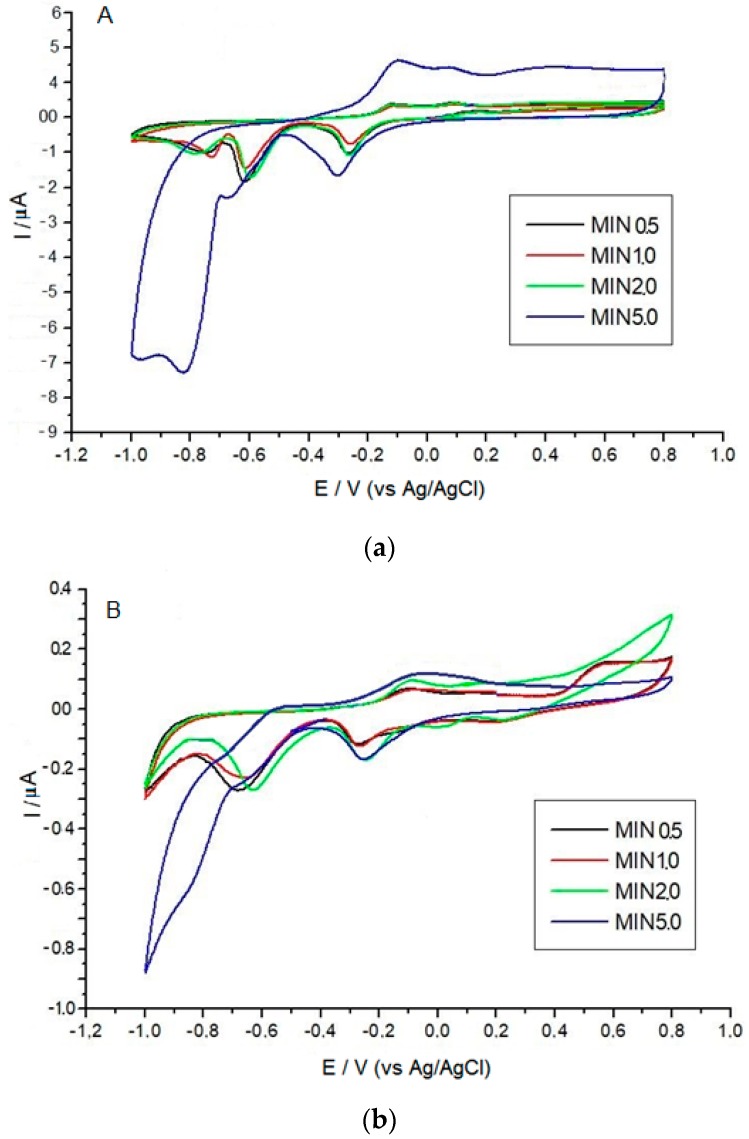
Effect of the immersion time of the CuO/Cu (**A**) and CuONf/Cu (**B**) electrodes in 0.1 mol·L^−1^ NaOH, in the cyclic voltammograms, at 100 mV·s^−1^.

Five CuO/Cu sensors and five CuONf/Cu sensors were fabricated according to the procedures already described. These sensors were used to check the reproducibility of measurements for successive COD determinations by measuring the voltammetric current signal of a concentration of COD (1000, mg·L^−1^ of O_2_) and checking the current over a period of ten days. Fifteen consecutive measurements were performed daily with each electrode. After finishing the fifteen measurements the sensors were thoroughly washed with distilled-deionized water and left exposed to the laboratory atmosphere until next day. The corresponding standard deviation values of the results are numerically and graphically represented in Figure 3C. Using the CuO/Cu sensor, the intraday relative standard deviation (RSD) is 12.26% for 15 determinations, suggesting poor reproducibility, while using the CuONf/Cu sensor the RSD values were 6.03%, indicating twice as much reproducibility for this electrode. After one week’s work, the current responses decreased 63.3% for the CuO/Cu sensors and 7.4% for the CuONf/Cu sensors. These values support three assumptions: (a) lack of stability of the CuO/Cu surface. Probably due to fouling or oxide accumulation; (b) good stability of the CuONf/Cu electrode. Probably due to the fact that the electrode becomes physically sturdier because the Nf film is firmly attached to the copper substrate minimizing fouling so that the active electrode surface remains practically unaltered during at least 15 consecutive determinations and (c) the fabrication method of the modified electrodes is appropriate and reproducible. To get some insight on the reasons behind the lack of reproducibility of the CuO/Cu electrode, the sensors were withdrawn from the solution, thoroughly rinsed with water and then used for experiments in a 0.1 mol·L^−1^ NaOH solution (Figure 4). Oxidation and reduction peaks characteristic of surface oxide formation on the electrode were observed to increase during the voltammetric scans. These peaks probably correspond to the formation of surface oxide, which increases with immersion time of the CuO/Cu electrode in the NaOH solution (Figure 4A). After 5 min immersion time, an approximately five times increase in the current with respect to the first cycle was observed. This observation confirmed surface formation of copper oxide, leading to an increase of the CuO/Cu electrode effective area. The gradual surface increment of the electrode could be one of the reasons for the higher sensitivity of this sensor, but, on the other hand, it could also be the reason for the irreproducible results observed during COD determination using this electrode. For the electrochemical determinations of COD in wastewaters, some particular interference should be taken under consideration. One of the most serious interference rises from the fouling of the electrode surface by dissolved organic matter adsorption [32,33,34]. The fouling process is better developed on an exposed metallic surface and that is why the CuO/Cu electrode is more affected by this type of interference than the Nafion-covered CuONf/Cu electrode. Experiments similar to the previously described for the CuO/Cu electrode did not show any significant alteration by oxide growth or fouling on the CuONf/Cu surface, Figure 4B. Modification of the electrode surface with the Nf membrane prevents formation of surface hydroxides which may contribute to the progressive increase of the surface area in the case of the CuO/Cu sensor. As indicated before, the properties of Nf as being an electroinactive, chemically inert, hydrophilic and water insoluble material together with its size-exclusion properties, make this polymer ideal for the purposes of protecting the electrode from fouling and allowing for its best analytical performance. 

### 3.4. Effect of NaOH Concentration

**Figure 5:**
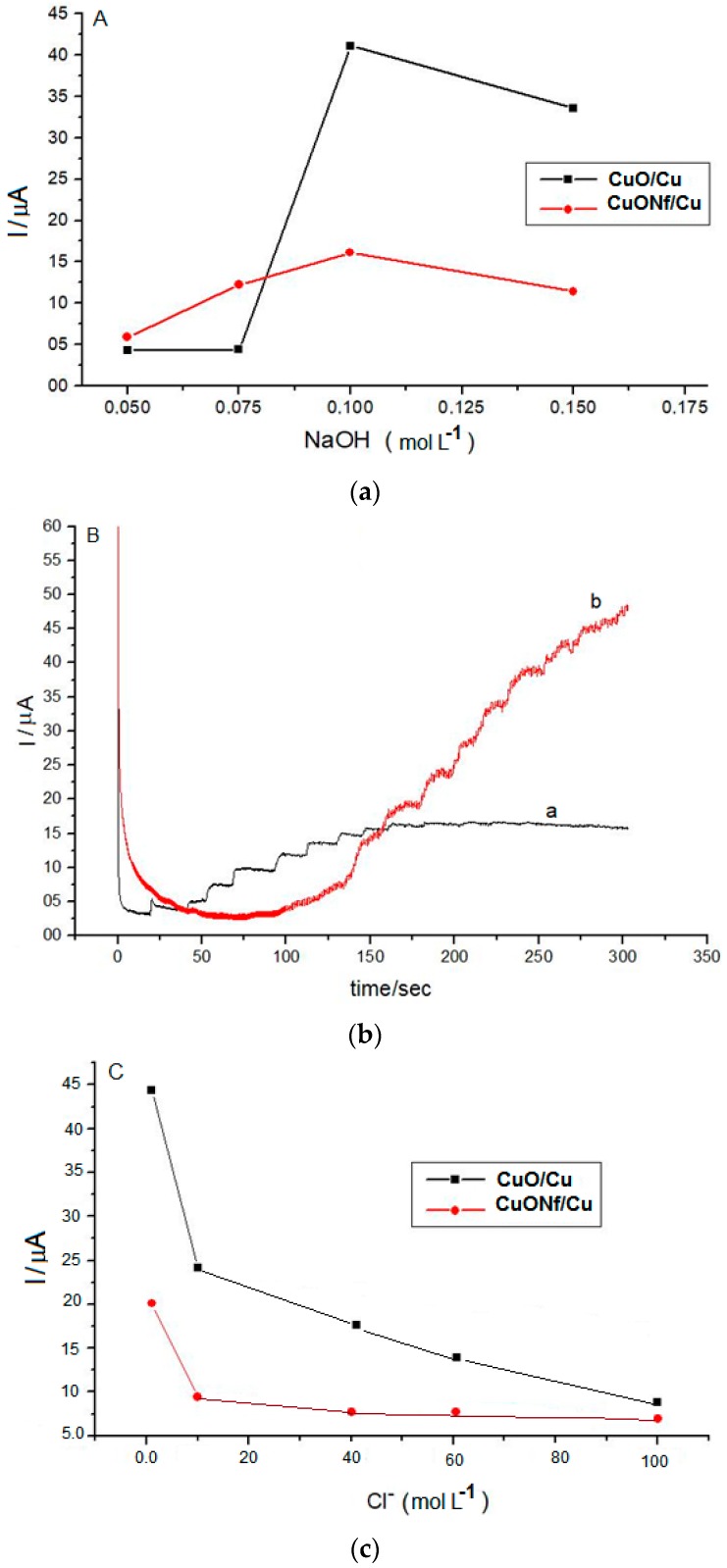
(**A**)Effect of NaOH on the electrocatalytic performance of the CuO/Cu electrode (black dots) and the CuONf/Cu electrode (red dots). (**B**) Amperometric response of CuO/Cu (black line) and CuONf/Cu (red line) electrodes to different concentrations of COD. Detected potential, 0.8 V. Electrolyte, 0.1 mol·L^−1^ NaOH solution. (**C**) Effect of Cl^−^ concentration on the electrocatalytic performance of the CuO/Cu (black dots) and the CuONf/Cu (red dots) electrodes.

An alkaline medium is required to enhance the electrocatalytic activity of metal oxides. Thus, the effect of NaOH concentration on the electrocatalytic performance of the CuO/Cu and CuONf/Cu sensors on glucose oxidation was tested in 0.05 to 0.15 mol·L^−1^ NaOH solutions. Increasing NaOH concentration resulted in an increase of the anodic current wave for both sensors, because glucose oxidation is more efficient at high NaOH concentration (Figure 5A). Using the CuO/Cu sensor, when the concentration of NaOH increases above 0.1 mol·L^−1^, a high background noise was obtained and lots of gas bubbles were observed on the surface of the CuO/Cu sensor, suggesting that oxygen evolution from water oxidation gradually becomes a more important reaction than oxidation of organic pollutants. The results were consistent with the previous experimental observations when an increased background current was observed at high NaOH concentrations. Results of our experiments indicated that, a 0.1 mol·L^−1^ NaOH solution was optimal for glucose oxidation using the CuONf/Cu sensor. Higher NaOH concentrations reduce the current to very low values.

### 3.5. Amperometric Detection of COD

Figure 5B, depicts the amperometric response of different concentrations of COD at CuO/Cu and CuONf/Cu sensors. The applied potential was 0.8 V, and the electrolyte 0.1 mol·L^−1^ NaOH solution. After the addition of glucose (400 mg·L^−^^1^ COD), well-defined oxidation current signals were observed at CuO/Cu and CuONf/Cu sensors. Background current decreases dramatically and attains a steady state at about 150 s at CuO/Cu (black line). The CuONf/Cu sensor (red line) had a quick response to the addition of samples after 100 s, while at CuO/Cu sensor the current signal loses the proportionality with the increase of the concentration of glucose in solution. According to the results obtained in above sections, this behaviour was attributed to the progressive increase of the area of the CuO/Cu sensor surface during measurements. Compared to the CuO/Cu sensor, it is apparent that the larger stability of CuONf/Cu in the DQO measurements could be attributed to the structure of Nf film preventing the formation of surface hydroxides. These hydroxides may contribute to the progressive increase of the area of the CuO/Cu sensor surface during measurements, which preclude reproducible DQO determination.

### 3.6. Interference of Chloride Ions

Chloride ions are one of the major factors affecting COD measurements when the dichromate titration method is used [9]. Many pure metals or metal-alloy electrodes usually become poisoned by chloride ions with the consequent loss of their activity [10]. Since chloride is a ubiquitous ion in most water systems, the effects of chloride ion concentration on the COD determination when using the CuO/Cu and CuONf/Cu sensors, was investigated (Figure 5C). For the conventional titration method, it is typical to precipitate the chloride ions by addition of Ag^+^ before COD determination to avoid chloride electrode poisoning. For our experiments, we chose a Cl^−^ concentration range from 10 to 100 mg·L^−1^, based on the usual Cl^−^ concentration level in domestic water (30–100 mg·L^−1^). Concentration of Cl^−^ ions between 10 to 40 mg·L^−1^ produced no significant effect on the COD determination using the CuONf/Cu sensor. It was necessary to reach 80 mg L^−1^ chloride ion to observe about 14% oxidation current decrease. However, using the CuO/Cu sensor Cl^−^ concentrations just above 10 mg·L^−1^ decreased the oxidation current about 72%. The decrease in current for the CuO/Cu sensor is due to the electrocatalytic oxidation of Cl^−^ on the electrode surface. This electrocatalytic process is hindered by the Nf film that covers the CuONf/Cu electrode surface. Based on these and the previous results, the analytical measurements leading to COD quantitation in some water samples were carried out using the CuONf/Cu sensor.

### 3.7. Analytical Figures of Merit

**Figure 6:**
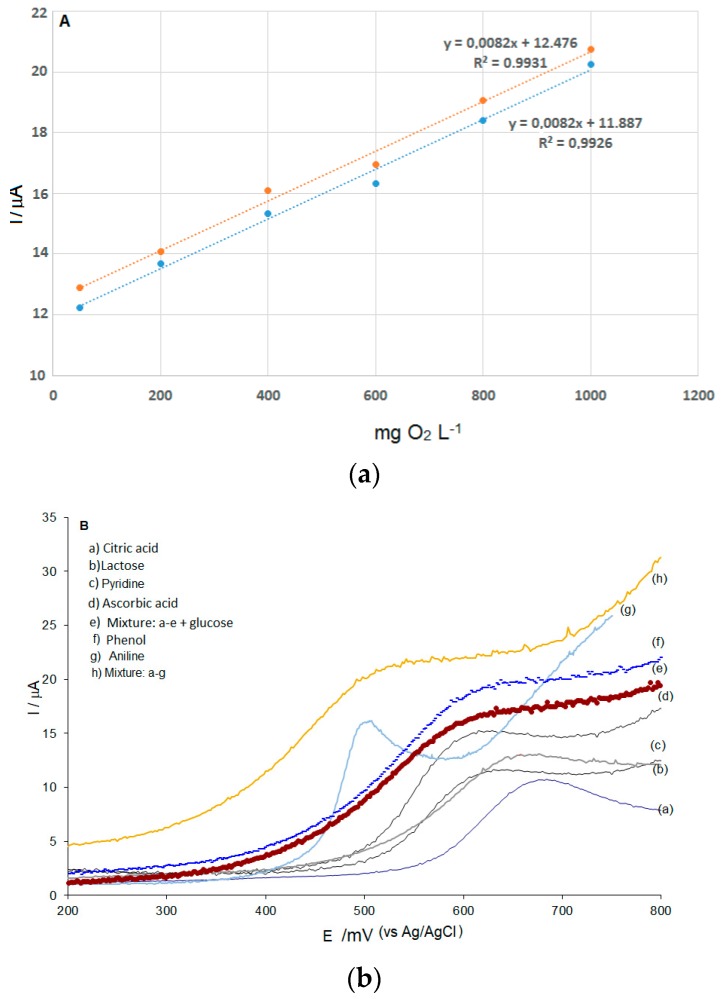
(**A**) Comparison of calibration curves for COD obtained by variation of Glucose concentration (blue dots) and a mixture of five organic compounds (orange dots), using the CuONf/Cu modified electrode. (**B**) Linear sweep voltammograms at 80 mV·s^−1^ of different organic compounds in a 0.1 mol·L^−1^ NaOH solution.

Under optimized conditions, linear range and detection limit were calculated using the CuONf/Cu sensor based on the curve of Figure 3B. It was found that the oxidation current signal is proportional to the concentration of glucose (COD C, mg·L^−1^ of O_2_) over the range from 50 to 1000 mg·L^−1^ as shown in the figure. The linear regression equation is: I / µA = 12.0516 + 9.17 × 10^−3^ x, R^2^ = 0.9957, (n = 6). Detection limit calculated based on the 3*s* criterion measuring a blank solution was 2.11 mg·L^−1^ (n = 6). To evaluate the linear range and the detection limit of the proposed method, seven organics with known theoretical COD values were selected as standard samples for calibrating the COD measurements (Figure 6A). The results showed that all organic compounds investigated were fitted into a linear equation: I / µA = 8.3 × 10^−3^ x + 12.48, R = 0.9981, (n = 6). Linear voltammograms were obtained for five different types of standard samples with the same COD value (Figure 6B). The relative standard deviation (RSD) for these measurements was 3.1 (n = 6). These results show that COD measurements by the proposed method were practically independent of the type of organic contaminant causing it. This fact stands for the use of glucose as a valid representative of organic contaminants in water systems, as carried out in this work, and validates its foundations for the quantitative COD determinations described. Based on the above findings, the accuracy of the proposed method was evaluated using a synthetic sample containing 90 mg·L^−1^ glucose as a standard COD test reagent. Linear voltammograms were obtained using a series of test solutions prepared in the same way. A 4.2 relative standard deviation (RSD) (n = 6) was obtained. The average result obtained after six replicate COD measurements was 91.04 ± 0.09 mg·L^−1^. Statistical analysis using the Student’s *t*-test showed that there is no statistical significant difference between the actual and found concentration at the 95% confidence. 

Compared to the reported electrodes shown in Table 1, the newly developed electrode exhibits good linear range, sensitivity, tolerance level towards Cl^−^ ions and lower detection limit. Moreover, this new sensor not only exhibits the electrochemical oxidation capacity of organic contaminants but also provide excellent chemical and physical resistance. 

### 3.8. Analytical Application

To check the performance of the CuONf/Cu electrode for analysis of real samples, various types of water samples obtained from Machala City (Ecuador), River water samples (Río Jubones), municipal slaughterhouse residual water samples (Pasaje City) and estuary Huayla water samples (Machala city), were analyzed by the conventional dichromate method and by the proposed electrochemical method (Table 2). This comparison showed that the proposed method was a valid alternative to determine COD concentration in real water samples. Relative errors of the COD determination were in the range 3.09% to 7.47%. Incorporation of known amounts of glucose in the water samples obtained from the Jubones River and determination of their COD concentration using the CuONf/Cu electrode produced recoveries around 103 to 105% (Table 3).

## 4. Conclusions

The analytical performance of a CuO/Cu electrode for COD determination was substantially improved by covering its surface with a Nf film. The resulting CuONf/Cu electrode, although around 15% less sensitive than the original CuO/Cu one, was more accurate, more precise and has longer useful lifetime. These improved characteristics were mainly due to the anti-fouling, and anti-passivation protective shield provided by the Nf film. Moreover, the Nf film helps in giving the electrode better physical and mechanical endurance thus it could be used, without noticeable performance deterioration for longer times than the electrode with no Nafion. Preparation of the CuONf/Cu electrode is relatively simple, fast and of low cost. Quantitation of the COD content of river, estuary and waste water samples using the electrochemical methodology developed for application of the new electrode, showed that the new method is as accurate and sensitive as the conventional one but far more rapid, less laborious, less expensive and greener. The CuONf/Cu sensor alleviates poisoning and displays good sensitivity and reproducibility in continuous COD determination with the possibility of being used for analysis of in flow-through cells. 

## Figures and Tables

**Table 1:** Analytical features of different electrochemical sensors for COD determination.

	Limit of Detection (mg·L^−1^)	Linear Range (mg·L^−1^)	Sensitivity	Tolerance Level Towards Cl^−^ Ions
No. 8	1.05	2–595	4 × 10^−4^ J·mg·L^−1^	0.1 mol·L^−1^
No. 9	20.3	53–2801	4 × 10^−4^ µA·mg^−1^·L^−1^	Not reported
No. 10	1.0	10–1533	0.14 µA·mg^−1^·L^−1^	1 mol·L^−1^
No. 11	1.1	10–1533	0.059 µA·mg^−1^·L^−1^	0.02·mol·L^−1^
No. 12	0.14	0.24–480	0.14 µA·mg^−1^·L^−1^	0.1·mol·L^−1^
No. 13	1.1	30–180	1.048 µA·mg^−1^·L^−1^	0.02·mol·L^−1^
No. 14	0.05	1–400	3.85 µA·mg^−1^·L^−1^	0.3·mol·L^−1^
No. 15	3.6	30–600	Not reported	2·mol·L^−1^
No. 18	0.05	1–400	3.85 µA·mg^−1^·L^−1^	0.3·mol·L^−1^
No. 19	0.609	1.92–768	0.08 µA·mg^−1^·L^−1^	0.02·mol·L^−1^
No. 20	20	50–2000	0.0022 µA·mg^−1^·L^−1^	< 600·mg·L^−1^
No. 21	4.3	5–1400	0.252 µA·mg^−1^·L^−1^	Not reported
This work	2.11	50–1000	9.17 × 10^−3^ µA·mg^−1^·L^−1^	10·mg·L^−1^

**Table 2:** Comparison of COD values (mg·L^−1)^ obtained by the proposed method with those obtained by the conventional dichromate method. n = 6, t (°C) = 25.

Sample	Proposed Method		Conventional Methods		Relative Error (%)
	COD (mg·L^−1^)	RSD (%) n = 3	COD (mg·L^−1^)	RSD (%) n = 3	
Water from Jubones River	22.21	0.22	24.00	0.09	7.47
Water from Municipal slaughterhouse	4305	0.04	4500	0.03	4.33
Estuary Huayla	1191	0.01	1500	0.1	3.09

**Table 3:** Results of the recovery experiment, %.

Sample	COD Initial (mg·L^−1^)	COD Added (mg·L^−1^)	COD Found (mg·L^−1^)	Recovery (%) *
Sample 1	24	300	339 ± 0.3	104.64 ± 3.0
Sample 2	24	300	335 ± 0.2	103.40 ± 4.0
Sample 3	24	300	340 ± 0.6	104.93 ± 3.0

* % R = COD found / (COD initial + COD added) × 100.
